# Immunomodulatory effects of *Lactiplantibacillus plantarum* CCFM8661 + stachyose on cyclophosphamide-induced immunosuppression mice

**DOI:** 10.3389/fimmu.2025.1513531

**Published:** 2025-01-27

**Authors:** Weiwei Ma, Hang Sun, Lian Lian, Lidong Guo, Yanyan Wang, Lili Huang

**Affiliations:** College of Pharmacy, Heilongjiang University of Chinese Medicine, Harbin, China

**Keywords:** immunoregulation, probiotics, *Lactiplantibacillus plantarum*, cyclophosphamide, intestinal microbiota

## Abstract

**Introduction:**

The increasing stress of modern life has led to a decline in immunity, sparking widespread interest in new strategies to boost immune function. *Lactiplantibacillus plantarum* and stachyose have gained attention for their immune-regulating effects, but the mechanisms of their combined application remain unclear. This study aims to investigate the immunoregulatory effects of *Lactiplantibacillus plantarum* CCFM8661 combined with stachyose in cyclophosphamide-induced immunocompromised mice.

**Methods:**

Mice were divided into the normal control group, model control group (normal saline), positive drug control group (levamisole hydrochloride, 10 mg/kg), and low, medium, and high-dose groups (1.5×10_5_, 1.5×10_6_, and 1.5×10_7_ CFU of *Lactiplantibacillus plantarum* CCFM8661 + 1.5 mg stachyose). Each treatment group received continuous oral gavage administration for 28 days. On days 23 and 24, except for the normal control group, all other groups were intraperitoneally injected with cyclophosphamide (40 mg/kg) to establish an immunosuppressed model. On day 28, 30 minutes after the final administration, the mice were euthanized to collect the thymus, spleen, serum, ileum, and feces for subsequent analysis of immune indicators, intestinal barrier function, serum cytokines, and intestinal microbiota.

**Results:**

The combination significantly improved immune organ atrophy, restored intestinal morphology, and normalized cytokine levels in immunosuppressed mice, indicating enhanced immune function. Additionally, it restored intestinal microbiota diversity by increasing the abundance of *Muribaculaceae* while reducing *Lachnospiraceae*, potentially promoting intestinal homeostasis.

**Discussion:**

The combination of *Lactiplantibacillus plantarum* CCFM8661 and stachyose has immune-enhancing effects, potentially achieved by regulating inflammation levels and maintaining intestinal homeostasis.

## Graphical Abstract

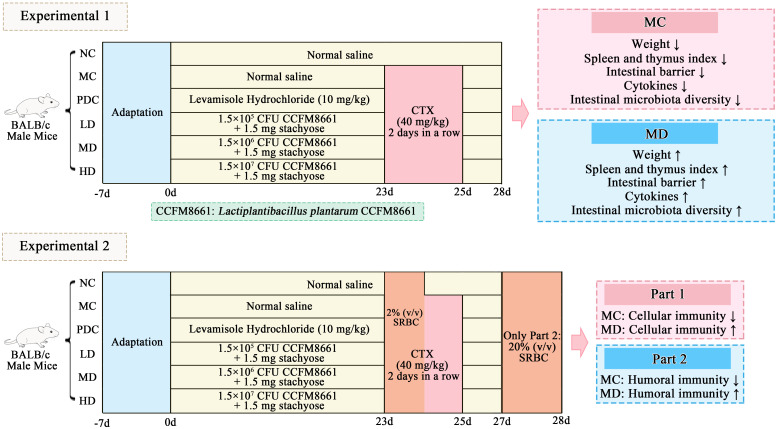

## 1 Introduction

The accelerating pace of modern life has led to increased stress, contributing to a notable decline in population immunity (1, 2). The COVID-19 pandemic, which emerged in 2019, resulted in hundreds of millions of infections and substantial mortality, underscoring the critical need for enhanced immune health (3, 4). Throughout this crisis, many individuals displayed diminished immunity (5, 6). This reality has prompted widespread interest in strategies to boost immune function and prevent disease. While traditional chemical immunomodulators have shown limited effectiveness against immunosuppressive disorders and often come with significant side effects, alternative therapeutic agents are gaining prominence (7). These include traditional Chinese medicine extracts (8, 9), nano-formulations (10), probiotics (11, 12), etc. Probiotics, as active microbial agents, enhance disease resistance by stimulating the immune system and improving immune responses (13). Additionally, they produce beneficial metabolites and help maintain the ecological balance of intestinal microbiota (14, 15).

The World Health Organization defines probiotics as “live microorganisms that, when administered in adequate amounts, confer a health benefit on the host” (16). Probiotic strains play a crucial role in immune regulation in various diseases, which may be related to their ability to modulate intestinal microbiota (17–19). In addition, the functions of probiotics in regulating cellular immune factors (20–22) and repairing the intestinal barrier (23) are also closely related to their role in enhancing immunity. In recent years, many strains of *Lactiplantibacillus plantarum* have been found to possess immune-regulating effects (23, 24). Ervina (25) et al. believe that *Lactiplantibacillus* sp. plays a crucial role in immune maintenance. Lactoferrin combined with *Lactiplantibacillus* can significantly enhance the immune response in kittens, increase immunoglobulin levels, and regulate inflammatory factors (26). Goya-Jorge (27) et al. revealed through the TripleSHIME^®^ system that Heyndrickxia coagulans combined with *Lacticaseibacillus casei* enhances the body’s immunity by regulating the intestinal microbiota, protecting the intestinal barrier, and other mechanisms.

*Lactiplantibacillus plantarum* CCFM8661 has previously been shown to mitigate toxicity induced by benzo (a) pyrene and effectively address the metabolic damage caused by heavy metals (28–30). Li (31) et al. suggested that a combination of probiotics containing *Lactiplantibacillus plantarum* CCFM8661 can alleviate antibiotic-associated diarrhea induced by ampicillin in mice. Our laboratory’s previous research has shown that *Lactiplantibacillus plantarum* CCFM8661 can effectively enhance both humoral and cellular immunity in cyclophosphamide-induced immunosuppressed mice. The underlying mechanisms for its immune-boosting effects may involve the regulation of cytokines, improvement of the intestinal barrier, and modulation of the intestinal microbiota.

Stachyose, a novel functional water-soluble oligosaccharide naturally present in plants, is noted for its low sweetness, low caloric content, and remarkable stability, which makes it ideal for improving food quality and developing functional foods (32). Previous studies have demonstrated that stachyose exhibits anti-inflammatory properties (33), alleviates intestinal colitis (34, 35), improving intestinal microbiota (36) and other effects. When combined with isoflavones, stachyose enhances bioavailability, thus improving hyperlipidemia and hyperglycemia (37), and it also promotes the absorption of tea polyphenols while protecting the liver (38). Wang (39) et al. found that stachyose combined with Fu brick tea polysaccharides and sheep whey protein could improve the immunity of immunocompromised mice. However, the effects of the combined application of *Lactiplantibacillus plantarum* CCFM8661 and stachyose on immune function have not been previously studied.

Cyclophosphamide (CTX) is a cytotoxic chemotherapy agent known to induce immune deficiency by inhibiting both humoral and cellular immunity in animals. It is widely utilized in the creation of animal models for immunosuppression (40). In this study, CTX was employed to establish an immunocompromised mouse model to investigate the effects of varying doses of *Lactiplantibacillus plantarum* CCFM8661 combined with water-soluble oligosaccharide on the immune function of CTX-induced immunocompromised mice, aiming to provide a further theoretical basis for the development and utilization of probiotics.

## 2 Materials and methods

### 2.1 Experimental materials

#### 2.1.1 Bacterial strain and culture

*Lactiplantibacillus plantarum* CCFM8661 was acquired from Hebei Yiran Biotechnology Co., Ltd., while stachyose was sourced from Anhui Zhongxinkang Pharmaceutical Co., Ltd. Prior to the experiment, both *Lactiplantibacillus plantarum* CCFM8661 and stachyose were kept in a - 80°C refrigerator. Our experimental design included low, medium, and high dose groups, consisting of 1.5×10^5^, 1.5×10^6^, and 1.5×10^7^ CFU of probiotics (*Lactiplantibacillus plantarum* CCFM8661) and 1.5mg stachyose, all prepared with physiological saline and activated prior to use.

#### 2.1.2 Animals

SPF-grade, BALB/c male mice aged 6 weeks were obtained from Liaoning Changsheng Biotechnology Co., Ltd. (Shenyang, China). The animals were maintained in a barrier environment within a temperature-controlled room (20 to 23°C) and humidity-controlled setting (30 to 60%), maintained on a 12:12-hour light-dark cycle, with free access to food and water. Total animal experiments stuck to the regulations outlined in the Management of Experimental Animals of Heilongjiang University of Traditional Chinese Medicine and received approval from the Animal Ethics Committee of Heilongjiang University of Traditional Chinese Medicine (ethical approval code: 2024032915).

### 2.2 Experimental methods

#### 2.2.1 Experimental 1 design

Following a 7-day acclimatization period, BALB/c mice were randomly allocated into various groups: the normal control group (NC), model control group (MC), positive drug control group (PDC), and groups receiving low, medium, and high doses of a combination of *Lactiplantibacillus plantarum* CCFM8661 and stachyose (LD, MD, HD), each consisting of 10 mice. The PDC group received levamisole hydrochloride (10 mg/kg, Taiyuan in Shanxi Province Pharmaceutical Co., Ltd., Shanghai, China) via gavage, while the LD, MD, and HD groups were administered *Lactiplantibacillus plantarum* CCFM8661 (1.5×10^5^, 1.5×10^6^, or 1.5×10^7^ CFU) combined with 1.5 mg stachyose by gavage. Mice in the NC and MC groups administered an equal volume of physiological saline daily via gavage for a duration of 28 days. Except for the NC group, all other groups received intraperitoneal injections of cyclophosphamide (40 mg/kg, Jiangsu Hengrui Medicine Co., Ltd., Shanghai, China) once daily on the 23rd and 24th day of intragastric administration of *Lactiplantibacillus plantarum* CCFM8661 + stachyose to establish the immunosuppressive model. Mice in the NC groups were administered an intraperitoneal injection of the same volume of normal saline. At the conclusion of the experiment, the immune organs of the mice were weighed for analysis, blood samples were collected and keptd at −80°C, ileal tissues were harvested for histopathological examination, and fresh feces were harvested in sterile conditions and keptd at −80°C for analysis of intestinal microbiota.

#### 2.2.2 Experimental 2 design

The experimental animals were divided into two groups, each containing 10 mice. On the 23rd day of administration, each mice was intraperitoneally injected with 0.2mL 2% (v/v) SRBC (R22395, Shanghai Yuanye Co., LTD., Shanghai, China.) for sensitization. Four days after immunization with 2% (v/v) SRBC, the first group of mice was injected subcutaneously with 20 μL of 20% (v/v) SRBC at the left hind toe. 24h later, the animals were sacrificed at the end of the last measurement. Mice in the second group were immunized with 2% (v/v) SRBC for 5 days and sacrificed after blood collection.

#### 2.2.3 Trends in weight gain

The starting body weight of the mice in all groups was measured at 8:30 AM on the first day of the experiment, after which the corresponding treatments were administered. The weights of the mice in all groups were then measured and documented at 9:00 AM every two days.

#### 2.2.4 Determination of immune organ index

The thymus and spleen of the experimental animals were dissected and weighed, after which the indices for both the thymus and spleen were calculated. The organ index was calculated as follows:


$$\text{Thymus or spleen index }\left(\text{mg}/\text{g}\right)=\frac{\text{Thymus}\;\text{or}\;\text{spleen}\;\text{mass}\;\left(\text{mg}\right)}{\text{Animal}\;\text{body}\;\text{weight}\;\left(\text{g}\right)}$$

#### 2.2.5 Delayed type hypersensitivity determination

The degree of delayed-type hypersensitivity was measured by plantar thickening method. On day 23, each mouse was intraperitoneally injected with 2% (v/v) SRBC 0.2 mL for sensitization. 4 days later, the thickness of the left hind toe was assessed, followed by a subcutaneous injection of 20 μL of 20% (v/v) SRBC at the same site (approximately 1×10^8 SRBC per mouse). After 24 hours, the thickness of the left hind toe was assessed again, and the average of three measurements at the same site was calculated. The difference in toe thickness before and after injection was used to indicate the degree of DTH response.

#### 2.2.6 Determination of serum hemolysin level

All mice immunized with 0.2 mL of 2% (v/v) SRBC via intraperitoneal injection on the 23rd day after oral administration. After 5 days, corresponding to a total of 28 days of gastric gavage, blood was collected from the eyes into a centrifuge tube. The samples were allowed to sit for about 1 hour, then centrifuged at 2,000 r/min for 10 minutes to separate serum, which was diluted with normal saline at a 1:100 ratio. The diluted serum was placed into a 96-well plate, followed by the sequential addition of 10% (v/v) SRBC and complemented guinea pig serum. After incubating the plate in a water bath maintained at 37°C for 30 minutes, the supernatant was centrifuged and transferred to a separate plate. Join Drabkin’s solution (R22785, Shanghai Yuanye Co., LTD., Shanghai, China.) and set the half hemolysis hole. Finally, the optical density of the mixed solution in each well was assessed by an automatic microplate reader. The amount of hemolysin was expressed as the half hemolytic value (HC_50_), which was calculated as follows:


$$Sample\;HC_{50}=\frac{Sample\;optical\;density\;value\;\times \;dilution}{Optical\;density\;of\;SRBC\;at\;half\;hemolysis}$$

#### 2.2.7 Histopathological analysis of ileum

Ileal tissues were preserved in 4% paraformaldehyde and kept 48 hours, before being embedded in paraffin (JB-P5, Wuhan Junjie Electronics Co., Ltd, Wuhan, China) and sectioned into 4-μm slices (RM2016, Leica, Shanghai, China). The sections were stained with hematoxylin-eosin after dewaxing, followed by photography under light and examination using a microscope (Nikon Eclipse Tzu Hsiang L, 100x magnification; Nikon, Tokyo, Japan).

#### 2.2.8 Serum level testing

After the final administration, the blood of the experimental animals was centrifuged (2000r/min) for serum extraction and frozen in a −80°C refrigerator for determination. The levels of serum IgA (MM-0055M1), IgG (MM-0057M1), IFN-γ (MM-0182M1), TNF-α (MM-0132M1), IL-2 (MM-0105M1), IL-6 (MM-0163M1) and IL-12 (MM-0701M1) were detected by ELISA kits (Jiangsu Meimian Industrial Co., Ltd., Yancheng, China).

#### 2.2.9 Metagenomic analysis

Total microbial DNA was extracted from the samples using the MagPure Soil DNA KF kit (D6356-F-96-SH, Magen, Shanghai, China). DNA concentration determination and integrity analysis were performed using a NanoDrop2000 spectrophotometer (Thermo Fisher Scientific, MA, USA) and agarose gel electrophoresis. Subsequently, DNA fragmentation and purification were conducted. The Qubit dsDNA HS Quantification Kit (Q32851, LifeTechnologies, California, USA) was used to detect concentrations, and the quality of library fragments was checked using an Agilent 2100 system (Agilent, California, USA). Library construction, sequencing, and data analysis were carried out by Shanghai Ouyi Biomedical Technology Co., Ltd.

#### 2.2.10 Statistical analysis

All data were analyzed using S*P*SS 26.0 statistical software, with results presented as mean ± SD. Differences between groups were assessed using one-way analysis of variance (ANOVA). *P*<0.05 was considered to indicate a statistically significant difference.

## 3 Results

### 3.1 Effect of probiotics on body weight in mice

**Figure 1:**
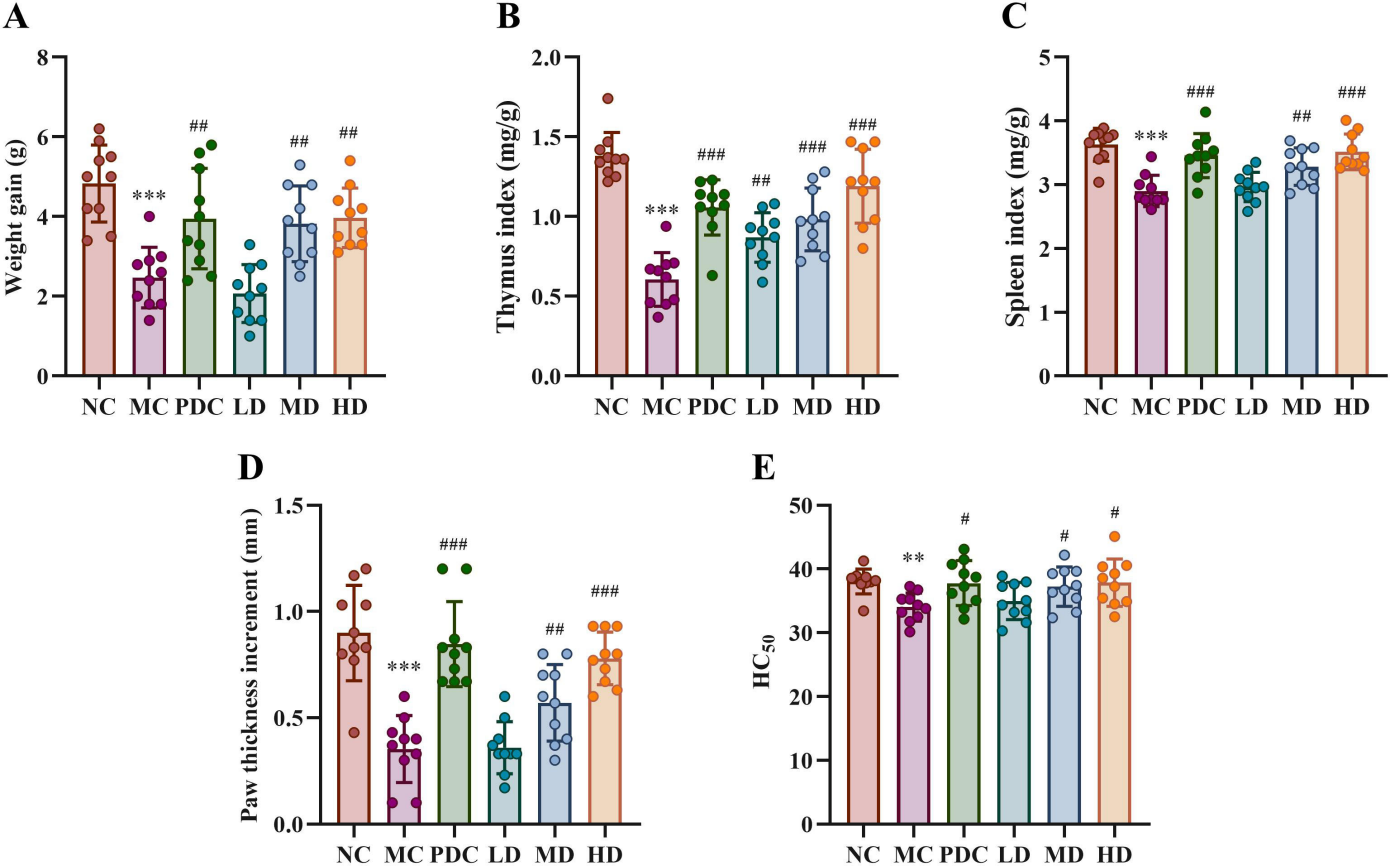
Effect of *Lactiplantibacillus plantarum* CCFM8661 + stachyose on immune-related indexes in immunocompromised mice model induced by cyclophosphamide. **(A)** Weight gain before and after the experiment in different drug administration groups; **(B, C)** Thymus index and spleen index; **(D)** Determination of cellular immune function by delayed type hypersensitivity; **(E)** Measurement of humoral immune function by serum hemolysin level. Normal control group: NC, model control group: MC, positive drug control group: PDC, *Lactiplantibacillus plantarum* CCFM8661 + stachyose-low dose groups: LD, *Lactiplantibacillus plantarum* CCFM8661 + stachyose-medium dose groups: MD, *Lactiplantibacillus plantarum* CCFM8661 + stachyose-high dose groups: HD. Data are presented as the mean ± SD. MC vs NC, * *P<*0.05, ** *P<*0.01, *** *P<*0.001; PDC, LD, MD. HD vs MC, ^#^ *P*<0.05, ^##^ *P*<0.01, ^###^ *P*<0.001, n=10.

**Figure 1A** illustrates that the difference in body weight before and after the experiment was statistically significant across the various administration groups (*P*<0.01). In comparison to the NC group, the weight gain in the MC group was markedly reduced (*P*<0.001), while the PDC, MD, and HD groups exhibited significantly increased weight gain compared to the MC group (*P*<0.01). These findings suggest that *Lactiplantibacillus plantarum* CCFM8661 + stachyose effectively mitigated weight loss induced by cyclophosphamide.

### 3.2 Effect of probiotics on thymus and spleen indices in mice

Compared to the NC group, the thymus index (**Figure 1B**) and spleen index (**Figure 1C**) in the MC group showed significant differences, with both indices decreasing markedly (*P*<0.001). The PDC group exhibited a significant increase in the thymus index (*P*<0.001), while the MD and HD groups also showed significant increases (*P*<0.01), with the high-dose group demonstrating a marked difference (*P*<0.001), when compared to the MC group. Cyclophosphamide-induced immunodeficiency can result in the atrophy of immune organs, whereas *Lactiplantibacillus plantarum* CCFM8661 + stachyose appears to mitigate the damage to these organs.

### 3.3 Effects of probiotics on cellular and humoral immunity in mice

As shown in **Figure 1D**, the foot thickness in the MC group was significantly reduced compared to the NC group (*P*<0.001). In comparison to the MC group, the PDC group exhibited a significant increase in foot thickness (*P*<0.001), while the MD and HD groups also showed significant increases (*P*<0.01), with the high-dose group demonstrating a marked difference (*P*<0.001). Additionally, regarding humoral immune function (**Figure 1E**), serum hemolysin levels in the MC group were lower compared to the NC group (*P*<0.01), while serum hemolysin levels in the PDC, MD, and HD groups increased following administration of the positive drug and adequate doses of *Lactiplantibacillus plantarum* CCFM8661 combined with stachyose (*P*<0.05). This indicated that *Lactiplantibacillus plantarum* CCFM8661+ stachyose was helpful in both cellular and humoral immunity of mice.

### 3.4 Effect of probiotics on intestinal barrier disruption

**Figure 2:**
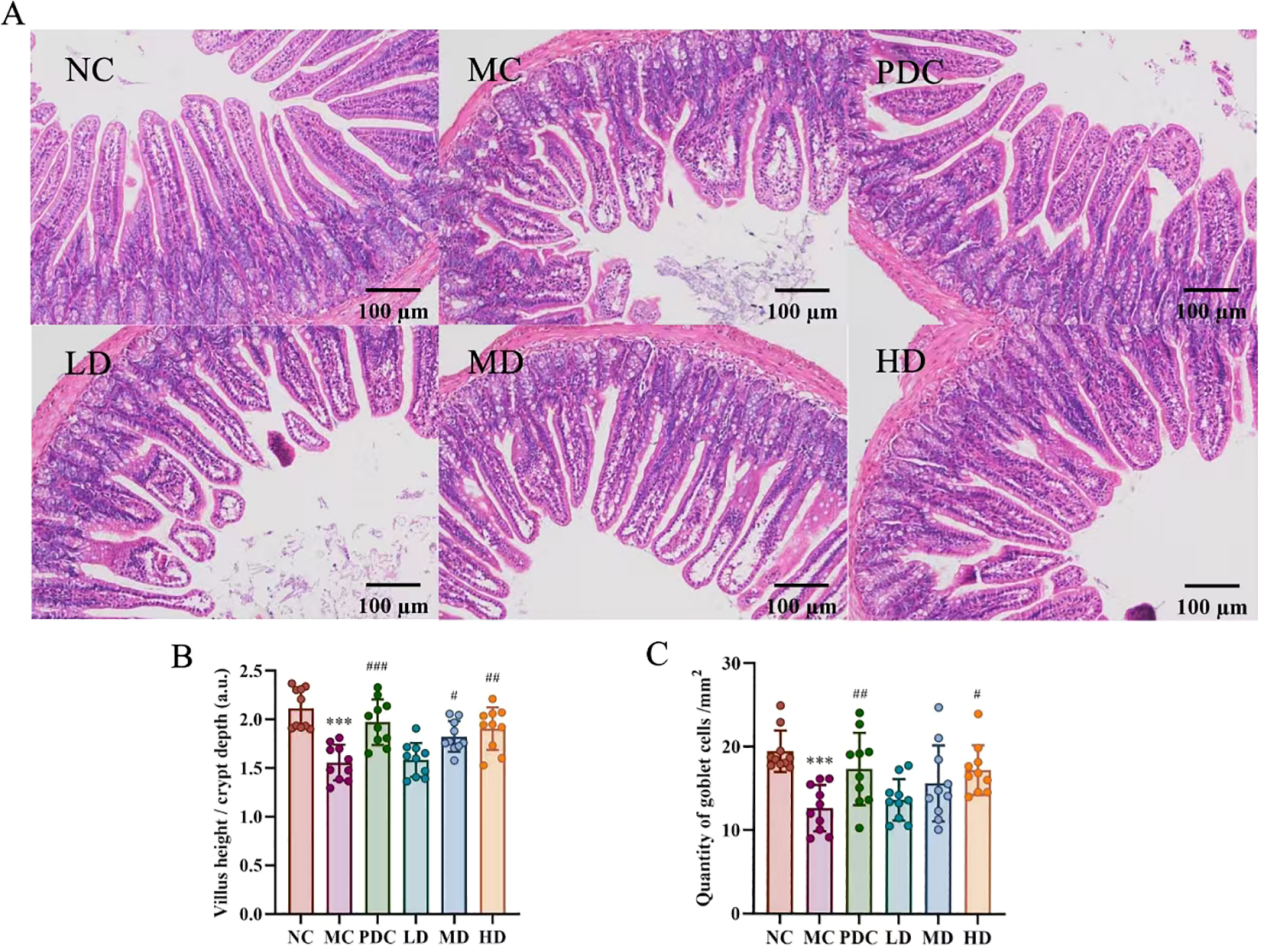
*Lactiplantibacillus plantarum* CCFM8661 + stachyose effect on the intestinal tract of immunocompromised mice induced by cyclophosphamide. **(A)** HE staining of mouse ileum sections (× 100); **(B)** Villus height/crypt depth; **(C)** The quantity of goblet cells in mice ileum per unit length. Normal control group: NC, model control group: MC, positive drug control group: PDC, *Lactiplantibacillus plantarum* CCFM8661 + stachyose-low dose groups: LD, *Lactiplantibacillus plantarum* CCFM8661 + stachyose-medium dose groups: MD, *Lactiplantibacillus plantarum* CCFM8661 + stachyose-high dose groups: HD. Data are presented as the mean ± SD. MC vs NC, * *P<*0.05, ** *P<*0.01, *** *P<*0.001; PDC, LD, MD. HD vs MC, ^#^ *P*<0.05, ^##^ *P<*0.01, ^###^ *P*<0.001, n=10.

The HE staining results of ileal tissues of mice in all categories are shown in **Figure 2A**. The small intestinal villi were tightly arranged and of uniform thickness in the NC group, while the MC group displayed atrophy and sparse villi. The PDC, MD, and HD groups showed alleviation of small intestinal villus lesions, with the PDC and HD groups demonstrating superior improvement compared to the MD group; the LD group had minimal effects. Villus height-to-crypt depth ratio (VH/CD ratio) was statistically considerably lower in the MC group by comparison with the NC group (*P*<0.001). Inversely, this ratio was increased in the PDC, MD, and HD groups in comparison with the MC group (*P*<0.05), with VH/CD ratio found to be higher in the HD group than in the MD group (**Figure 2B**). In Furthermore, the volume of goblet cells per the length of the unit was dramatically reduced in the MC group with respect to the NC group *(P*<0.001), whereas the volume of goblet cells was increased in the PDC group and the HD group with respect to the MC group (*P*<0.05) (**Figure 2C**). Altogether, these observations indicate that *Lactiplantibacillus plantarum* CCFM8661 + stachyose can effectively mitigate intestinal damage inflicted by cyclophosphamide in immune-impaired mice.

### 3.5 Effect of probiotics on serum cytokine levels in mice

**Figure 3:**
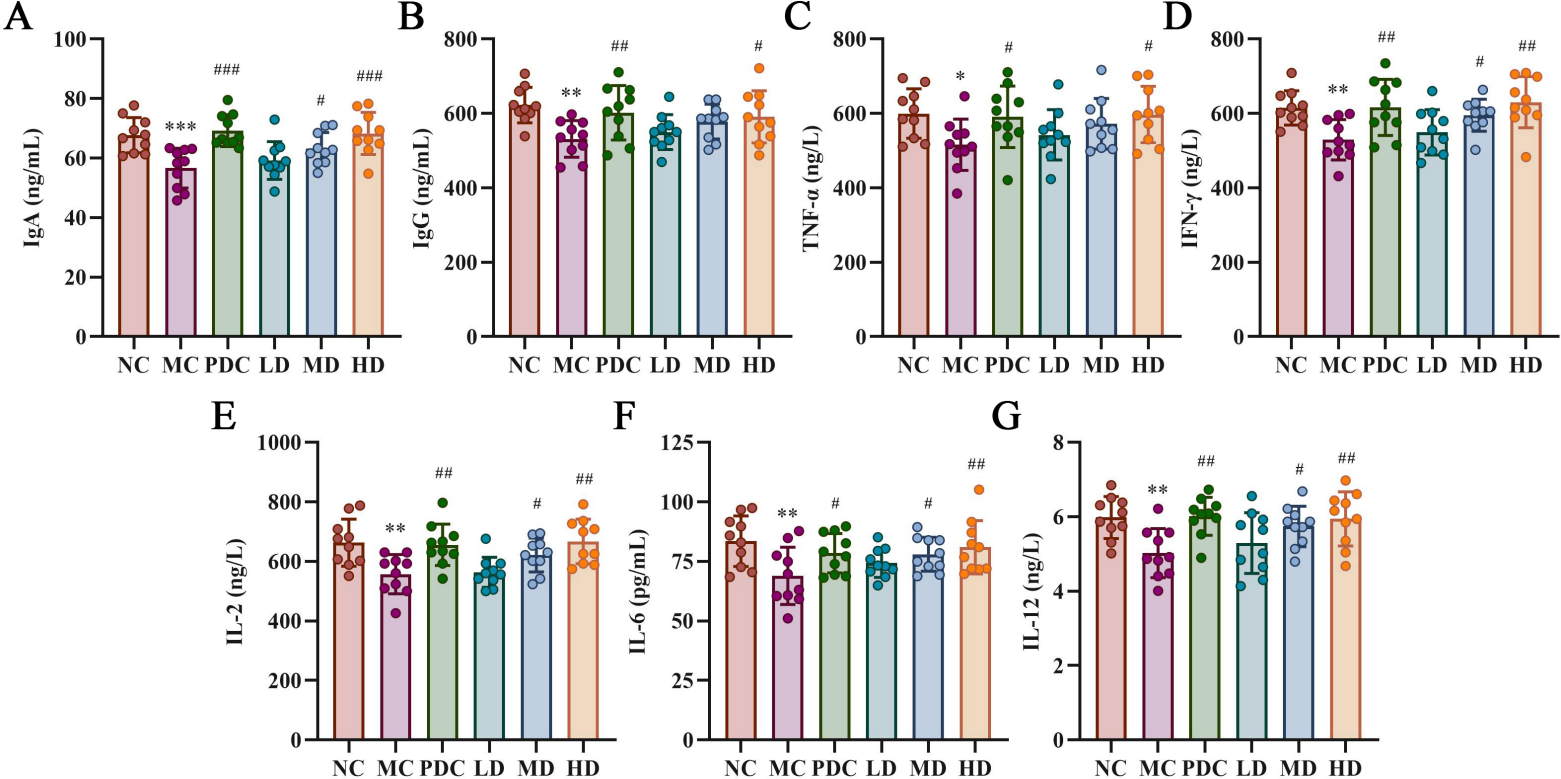
*Lactiplantibacillus plantarum* CCFM8661 + stachyose effect on cytokine levels in serum of immunocompromised mice induced by cyclophosphamide. **(A)** IgA; **(B)** IgG; **(C)** TNF-α; **(D)** IFN-γ; **(E)** IL-2; **(F)** IL-6; **(G)** IL-12. Normal control group: NC, model control group: MC, positive drug control group: PDC, *Lactiplantibacillus plantarum* CCFM8661 + stachyose-low dose groups: LD, *Lactiplantibacillus plantarum* CCFM8661 + stachyose-medium dose groups: MD, *Lactiplantibacillus plantarum* CCFM8661 + stachyose-high dose groups: HD. Data are presented as the mean ± SD. MC vs NC, * *P<*0.05, ** *P<*0.01, *** *P<*0.001; PDC, LD, MD. HD vs MC, ^#^ *P*<0.05, ^##^ *P*<0.01, ^###^ *P*<0.001, n=10.

**Figure 3** illustrates that the levels of IgG, TNF-α, IFN-γ, IL-2, IL-6, and IL-12 were obviously lower in the MC group versus the NC group (*P*<0.05), with IgA showing a marked decrease (*P*<0.001). In contrast, the PDC group exhibited increased levels of IgG, TNF-α, IFN-γ, IL-2, IL-6, and IL-12 compared to the MC group (*P*<0.05), and IgA levels were also obviously elevated (*P*<0.001). The LD group showed no appreciable change by comparison with the MC group, whereas the level of IgA, IFN-γ, IL-2, IL-6, and IL-12 were augmented in the MD group (*P*<0.05). The HD group demonstrated substantially increased levels of IgG, TNF-α, IFN-γ, IL-2, IL-6, and IL-12 in comparison with the MC group (*P*<0.05), as were the levels of IgA (*P*<0.001).

### 3.6 Effects of probiotics on intestinal microbiota in mice

#### 3.6.1 Alpha diversity analysis

**Figure 4:**
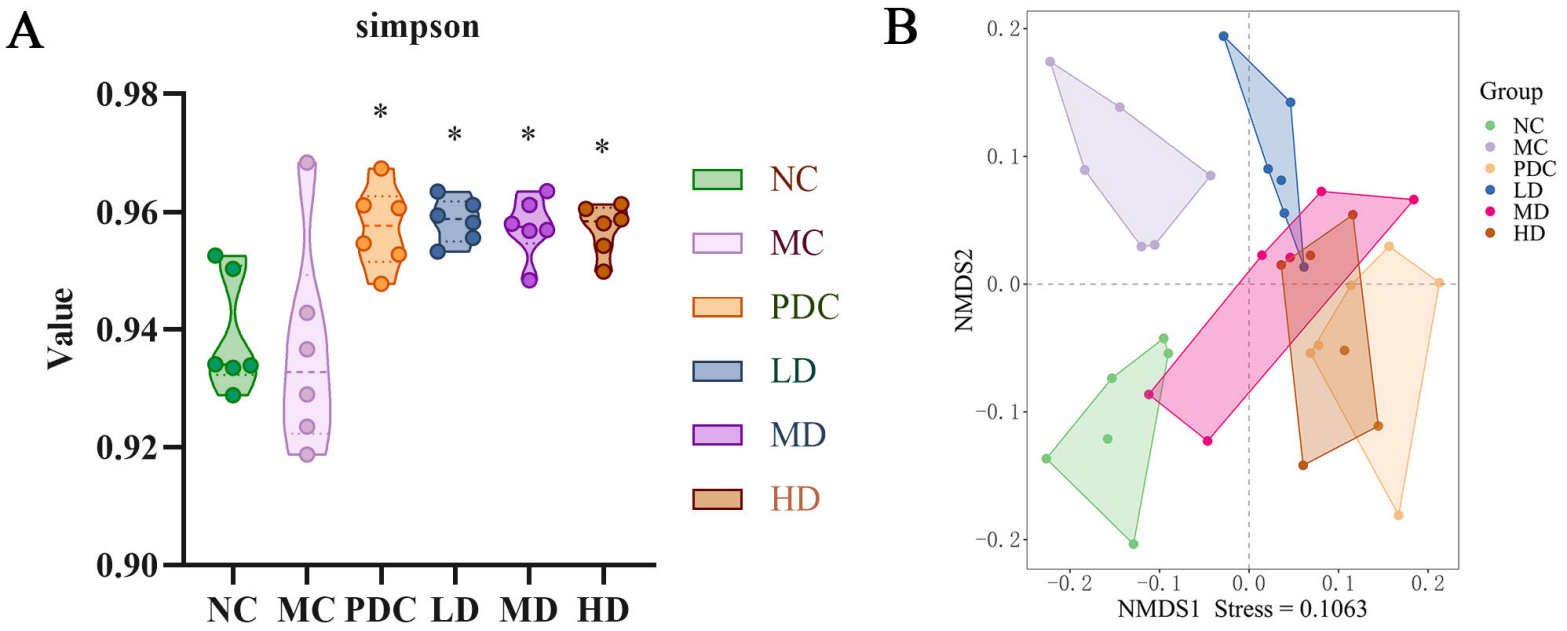
Effect of *Lactiplantibacillus plantarum* CCFM8661 + stachyose on the diversity of intestinal microbiota in cyclophosphamide-induced immunocompromised mice. **(A)** Simpson; **(B)** NMDS; Normal control group: NC, model control group: MC, positive drug control group: PDC, *Lactiplantibacillus plantarum* CCFM8661 + stachyose-low dose groups: LD, *Lactiplantibacillus plantarum* CCFM8661 + stachyose-medium dose groups: MD, *Lactiplantibacillus plantarum* CCFM8661 + stachyose-high dose groups: HD. Data are presented as the mean ± SD. MC vs NC; PDC, LD, MD. HD vs MC, * *P<*0.05, ** *P<*0.01, *** *P<*0.001; n=6.

Alpha diversity analysis assessed the multiplicity of intestinal microbiota across groups of mice, employing the Simpson index for evaluation, where a higher index signifies greater community diversity. No marked variation was observed between the NC and MC groups; however, when compared to the PDC group and various dose groups, the Simpson index significantly increased after administration (*P*<0.05). These results indicate that *Lactiplantibacillus plantarum* CCFM8661 + stachyose effectively enhance the multiplicity of intestinal microbiota in mice (**Figure 4A**).

#### 3.6.2 Beta diversity analysis

Alpha diversity reflects both the richness and uniformity of species within a sample. Beta diversity, on the other hand, is used to analyze striking discrepancies in the structure of microbial communities between different samples (or groups). In the NMDS analysis based on Bray-Curtis (**Figure 4B**), distinct characteristics of intestinal microbiota distribution were observed among the different mouse groups, with a stress value of 0.1063. Intestinal microbiota of the NC and MC groups differed significantly, with the LD group being more similar to the MC group, while the Group PDC, MD, and HD had microbial communities closer to the Group NC and away from Group MC. These results suggested that *Lactiplantibacillus plantarum* CCFM8661 + stachyose at medium and high doses could effectively modulate the composition of the immunodeficient mice intestinal microbiota to be closer to that of normal mice.

#### 3.6.3 Effect of probiotics on intestinal microbiota composition in mice

Based on species richness data, cumulative bar charts depicting the comparative richness of species at the phylum and species level were generated to visualize the diversity of different species across the groups at various taxonomic levels.

**Figure 5:**
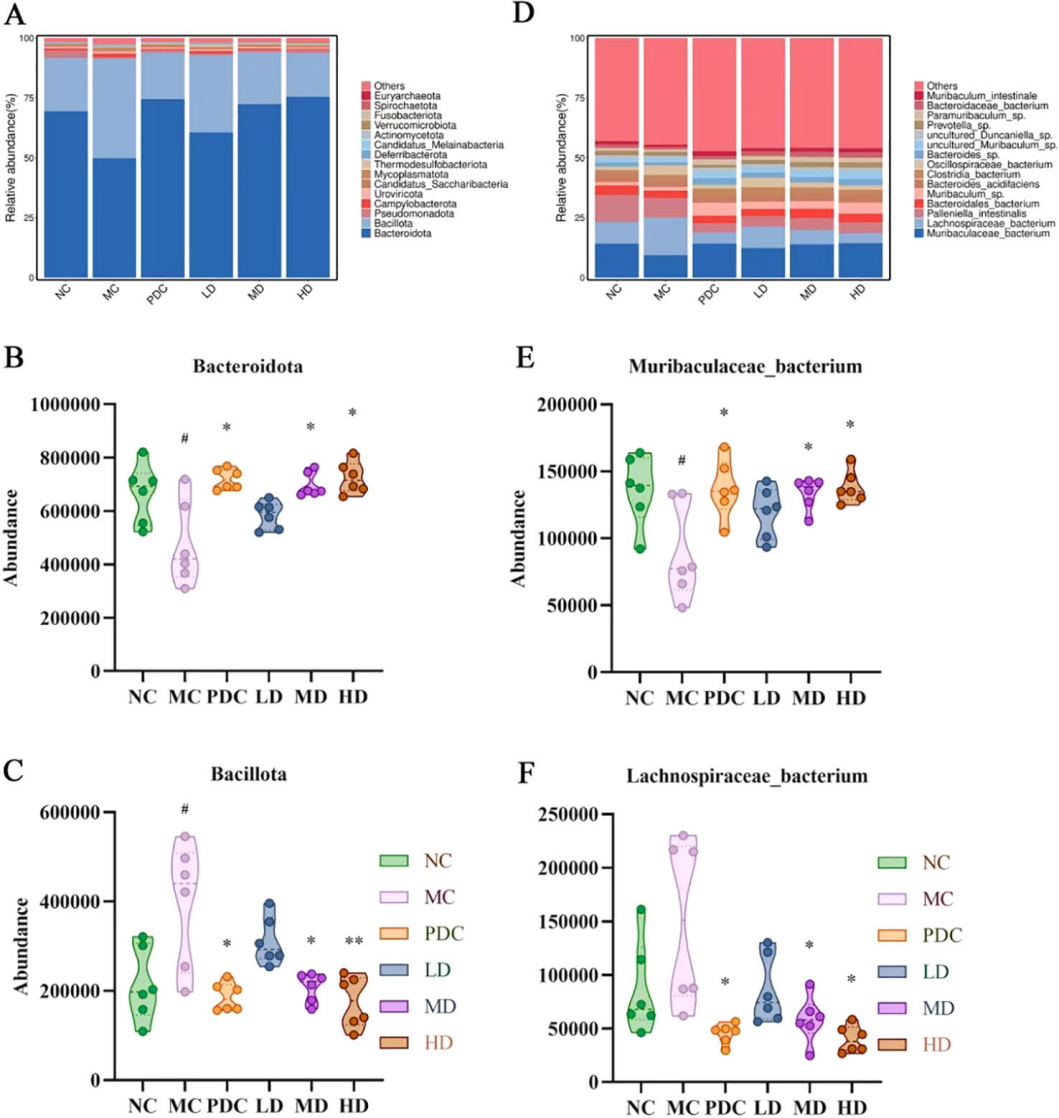
*Lactiplantibacillus plantarum* CCFM8661 + stachyose effect on the intestinal microbiota composition in immunocompromised mice model induced by cyclophosphamide. **(A)** Phylum level composition of intestinal microbiota; **(B)** Species level composition of intestinal microbiota; **(C)** *Bacteroidota*; **(D)** *Bacillota*; **(E)** *Muribaculaceae bacterium*; **(F)** *Lachnospiraceae bacterium*; Normal control group: NC, model control group: MC, positive drug control group: PDC, *Lactiplantibacillus plantarum* CCFM8661 + stachyose-low dose groups: LD, *Lactiplantibacillus plantarum* CCFM8661 + stachyose-medium dose groups: MD, *Lactiplantibacillus plantarum* CCFM8661 + stachyose-high dose groups: HD. Data are presented as the mean ± SD. MC vs NC; PDC, LD, MD. HD vs MC, * *P<*0.05, ** *P<*0.01, *** *P<*0.001; n=6.

Within the phylum level, *Bacteroidota* and *Bacillota* emerged as the predominant species across all groups, collectively accounting for over 90% of the total species. The relative abundance of *Bacteroidota* decreased by 19.57% (*P*<0.05) in the MC group over the NC group, while the relative abundance of *Bacillota* increased by 19.17% (*P*<0.05). Our results indicate a shift in the composition of the intestinal microbiota in immunocompromised mice induced by cyclophosphamide, characterized by an excessive increase in *Bacillota* and a corresponding inhibition of *Bacteroidota*, resulting in an elevated *Bacillota/Bacteroidota* ratio, which was consistent with the composition changes of intestinal microbiota in immunocompromised mice previously and was a manifestation of intestinal microbiota imbalance in mice (41). Notably, the relative abundance of *Bacteroidota* in the PDC, MD, and HD groups increased by 24.68% (*P*<0.05), 22.58% (*P*<0.05), and 25.62% (*P*<0.05), respectively, with respect to the MC group. Conversely, the relative abundance of *Bacillota* decreased by 22.14% (*P*<0.05), 19.83% (*P<*0.05), and 23.19% (*P*<0.01), leading to a reduced *Bacillota/Bacteroidota* ratio (**Figures 5A–C**). These results suggest that the combination of *Lactiplantibacillus plantarum* CCFM8661 and stachyose can significantly alter the relative abundance of phyla in the intestinal microbiota and mitigate the effects of cyclophosphamide.

At the species level, the organization of the groups of microorganisms consisted mainly included *Muribaculaceae bacterium*, *Lachnospiraceae bacterium*, *Palleniella intestinalis*, *Bacteroidales bacterium*, *Bacteroides acidifaciens*, *Clostridia bacterium*, *Oscillospiraceae bacterium* and *Bacteroides* sp. Versus the NC group, *Palleniella intestinalis*, *Bacteroidales bacterium*, *Bacteroides acidifaciens*, and *Bacteroides* sp. in the Group MC decreased. The relative abundance of *Muribaculaceae bacterium* was diminished by 4.84% (*P*<0.05). In Group PDC, MD, and HD, the relative abundance of *Muribaculaceae bacterium*, *Muribaculum* sp., *Bacteroides acidifaciens* and *Bacteroides* sp. enhanced compared to Group MC. The relative abundance of *Muribaculaceae bacterium* was elevated by 4.86% (*P*<0.05), 4.53% (*P*<0.05), and 5.05% (*P*<0.05), respectively. Within MC group, the relative abundance of *Lachnospiraceae bacterium*, *Clostridia bacterium*, and *Oscillospiraceae bacterium* was elevated compared to the NC group. The relative abundance of *Lachnospiraceae bacterium* increased by 6.67%. In the PDC, MD, and HD groups, the relative abundance of *Lachnospiraceae bacterium*, *Palleniella intestinalis*, *Clostridia bacterium*, and *Oscillospiraceae bacterium* decreased with respect to the MC group. The relative abundance of *Lachnospiraceae bacterium* decreased by 11.01% (*P*<0.05), 9.62% (*P*<0.05), and 11.51% (*P*<0.05), respectively (**Figures 5D–F**). The combination of *Lactiplantibacillus plantarum* CCFM8661 and fructose significantly altered the relative abundance of each species among different groups of mice, as demonstrated by these results.

#### 3.6.4 Correlation analysis of dominant flora at the species level with immune-related indicators, intestinal barrier-related indicators and cytokines

**Figure 6:**
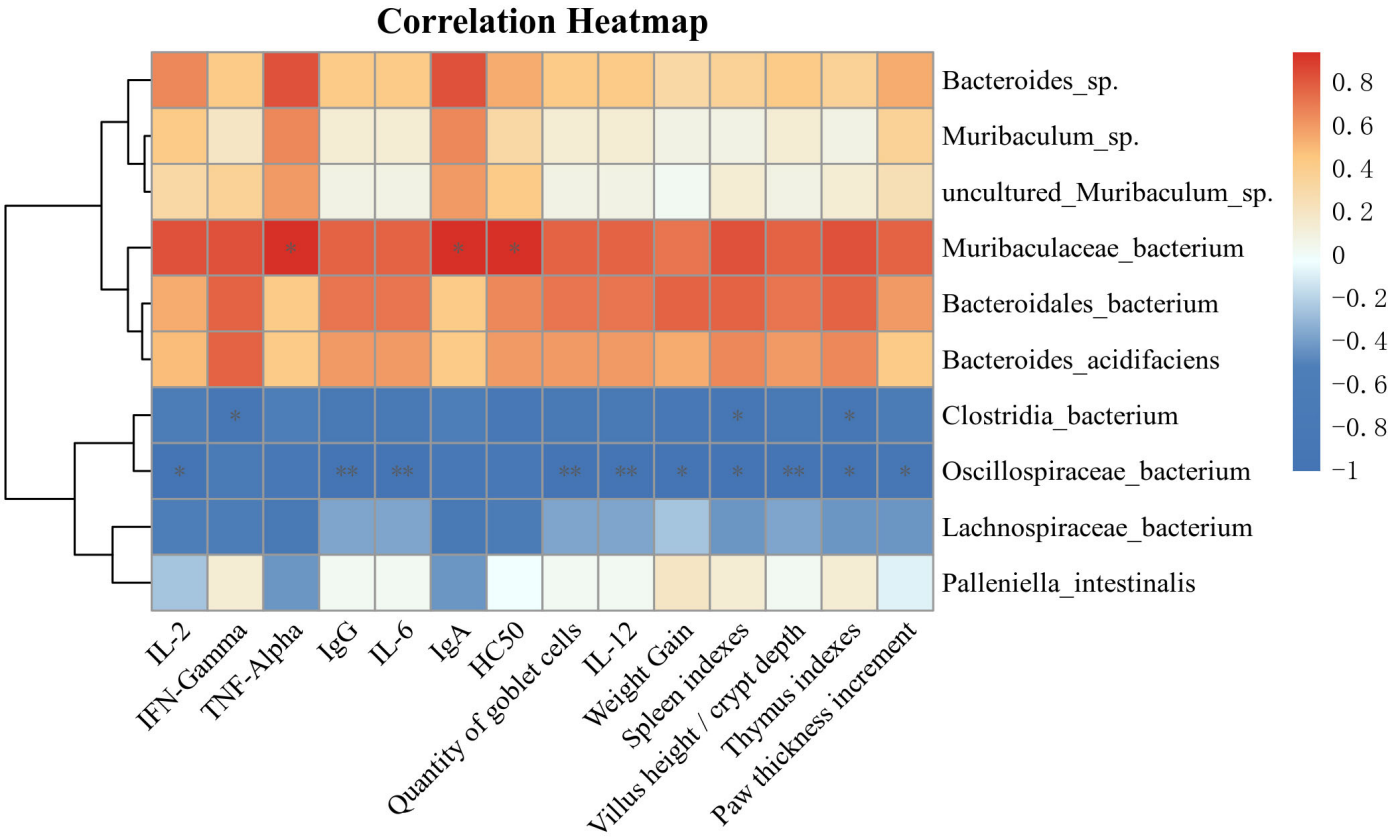
The Spearman method was employed to examine the correlations among immune-related indicators, intestinal barrier metrics, cytokines, and the expression of predominant bacteria within the intestinal microbiota. *, ** and *** (*P<*0.05, *P*<0.01 and *P*<0.001, respectively) indicate significant associations.

Spearman’s method was utilized to analyze the association of immune-related indices, intestinal barrier-related indices, cytokines with the expression of dominant species of intestinal microbiota at the species level, to explore the role of intestinal microbiota in the treatment process mediated by *Lactiplantibacillus plantarum* CCFM8661 + stachyose. The relative abundance of *Muribaculaceae bacterium* was dramatically and positively associated with HC50 and cytokines (IgA, TNF-α), and positively correlated with other immune indicators and intestinal barrier-related indicators, but not significantly. *Bacteroidales bacterium* was positively correlated with weight gain, immune organ index, and IFN-γ. *Oscillospiraceae bacterium* was significantly negatively correlated with weight gain, immune organ index, cellular immunity index, foot swelling degree, intestinal barrier-related indicators, and cytokines (IgG, IL2, IL-6, and IL-12). *Clostridia bacterium* was negatively correlated with immune organ index and IFN-γ (**Figure 6**).

## 4 Discussion

The study demonstrates that the combination of *Lactiplantibacillus plantarum* CCFM8661 and stachyose exhibits significant immunoregulatory potential. This combination effectively alleviates immune dysfunction by reducing immune organ atrophy, enhancing humoral and cellular immunity, restoring intestinal barrier integrity, and rebalancing the intestinal microbiota. The positive control drug, levamisole hydrochloride, exhibits significant immunomodulatory and immune-enhancing effects (42–44). The immunomodulatory capacity demonstrated by the combination of *Lactiplantibacillus plantarum* CCFM8661 and stachyose is comparable to that of levamisole hydrochloride. The findings highlight the ability of the innovative combination of *Lactiplantibacillus plantarum* CCFM8661 and stachyose to boost immune function and promote intestinal health.

As key immune organs, the thymus and spleen play critical roles in regulating the body’s immune function, and the thymus index and spleen index have become widely recognized visual indicators of immune status (45). The combination of *Lactiplantibacillus plantarum* CCFM8661 and stachyose can significantly alleviate the atrophy of the thymus and spleen in immunosuppressed mice. Notably, this study found that the thymus is more responsive to the dosage of this combination compared to the spleen, as improvements in thymic atrophy were observed even at low doses. Consistent with this study, certain *Lactiplantibacillus plantarum* strains have been shown to enhance the function of the thymus and spleen (46, 47). Zhang (48) et al. found that a combination of multiple prebiotics, including stachyose, and *Limosilactobacillus fermentum* DALI02 could restore immune organ indices in a CTX-induced immunosuppression model. It is worth noting that You (49) et al. reported that *Lactiplantibacillus plantarum* P101 had limited effects on reducing weight loss and immune organ atrophy. This discrepancy may be attributed to the irreversible damage caused by prolonged CTX injection to immune organs, making it difficult for *Lactiplantibacillus plantarum* to restore them.

Cellular immunity and humoral immunity are the two main defense mechanisms of the immune system. Immune function regulation can be assessed via DTH for cellular immunity (50) and serum hemolysin levels for humoral immunity (51). The combination of *Lactiplantibacillus plantarum* CCFM8661 and stachyose can significantly improve the specific immune function of immunosuppressed animals and exhibits a certain dose-dependent effect. Research by Zhang (52) et al. demonstrated that a specific dose of *Lactiplantibacillus plantarum* BF_15 could protect mice from a reduction in paw swelling and serum hemolysin antibody levels, thereby enhancing cellular and humoral immune function in mice. Zeng (53) et al. found that oral administration of *Lactiplantibacillus plantarum* expressing aCD11c can regulate cellular and humoral immunity, as well as enhance mucosal immunity. Studies have also shown that *Lactiplantibacillus plantarum* enhances cellular and humoral immunity in Paralichthys olivaceus (54, 55).

The integrity of the intestinal barrier is essential for maintaining overall health and is influenced by several key factors (56).Goblet cells maintain the mucosal barrier by producing mucin (57), while a higher villus height (VH) to crypt depth (CD) ratio indicates enhanced epithelial renewal, nutrient absorption, and barrier function (58, 59). The combination of *Lactiplantibacillus plantarum* CCFM8661 and stachyose can significantly counteract the damage to intestinal barrier integrity caused by immunosuppressants. Rawling (60) et al. found that a probiotic combination of *Lactiplantibacillus helveticus* and *Lactiplantibacillus helveticus* enhanced the intestinal barrier function and innate immunity of healthy zebrafish, with its mechanism linked to the growth of intestinal villi. Furthermore, Ren (61) et al. found that the combination of stachyose and *Lactiplantibacillus rhamnosus* GG improved intestinal barrier dysfunction caused by acute hypoxic hypoxia by mitigating inflammatory responses and oxidative stress. Zeng (62) et al. suggested that *Lactiplantibacillus plantarum* enhances intestinal immune function by regulating the intestinal barrier in an immunosuppressed mouse model, as evidenced by an increased VH/CD ratio and a higher number of goblet cells.

Immunoglobulins (IgA and IgG) are key markers of humoral immunity, reflecting B cell function (50, 63). Cytokines such as IL-2, IL-6, and IL-12 play vital roles in immune regulation and are associated with the secretion of IFN-γ and TNF-α (63–68). The combination of *Lactiplantibacillus plantarum* CCFM8661 and stachyose can counteract the reduction of cytokines caused by immunosuppressants. Cheng (69) et al. reported that cyclophosphamide disrupts cytokine secretion, a conclusion that was also supported by Xie et al. (70). Research by Meng (71) et al. revealed that *Lactiplantibacillus plantarum* KLDS1.0318 significantly upregulated the levels of various cytokines, effectively restoring abnormal cytokine levels in CTX-induced immunosuppressed mice. Similarly, Kim (47) et al. found that *Lactiplantibacillus plantarum* 200655 exhibited a similar trend in regulating IL-6 and TNF-α. Research has also demonstrated that the combination of stachyose, sheep whey protein, and Fu brick tea polysaccharides significantly upregulated the levels of IL-2, IL-6, and IgG (39). However, it has also been shown that *Lactiplantibacillus plantarum* can alleviate dextran sulfate sodium (DSS)-induced colitis in mouse models by reducing the release of pro-inflammatory cytokines (72, 73). These findings suggest that the regulatory effect of *Lactiplantibacillus plantarum* on cytokines is not a simple increase or decrease but rather a modulation of cytokine levels to restore them to a normal physiological state.

Previous studies have found that intestinal microbiota can induce the activation of immune cells, thereby influencing cytokine production, while immune cells can also mediate the neurogenic response of the intestinal microbiota through cytokines (74, 75). The combination of *Lactiplantibacillus plantarum* CCFM8661 and stachyose significantly improved the reduction in intestinal microbiota species diversity in immunocompromised mice. It regulated the composition and abundance of intestinal microbiota at various levels, thereby restoring intestinal ecological balance. At the phylum level, an imbalance between *Bacillota* and *Bacteroidota* was evident, with an increased *Bacillota*/*Bacteroidota* ratio, a pattern commonly linked to cyclophosphamide-induced immunodeficiency (76). Restoring the balance between these phyla may be a key indicator of improved immune function. *Lactiplantibacillus plantarum* CCFM8661 and stachyose restored the imbalance of the *Bacillota*/*Bacteroidota* ratio. At the species level, changes in various bacterial strains were observed in the immunosuppressed model, notably a significant decrease in *Muribaculaceae bacterium* and an increase in *Lachnospiraceae bacterium*. The combination of *Lactiplantibacillus plantarum* CCFM8661 and stachyose modulated the abundance of these bacteria, gradually restoring them to normal levels in a dose-dependent manner. Consistent with previous studies, *Muribaculaceae bacterium* and *Lachnospiraceae bacterium* play important roles in immune function. Chen (77) et al. found that *Muribaculaceae bacterium* was significantly down-regulated after the imbalance of immune-driven flora in immunodeficient mice. Previous studies have shown that sea cucumber tendon polysaccharide (SCTPII) can enhance immunity by regulating the diversity of intestinal microbiota. In the process of regulation, the homeostasis of intestinal microbiota is improved by reducing the relative abundance of *Lachnospiraceae* NK4A136 group (78).

However, this study still has some limitations. First, the potential effects of stachyose on the growth, viability, and metabolism of *Lactiplantibacillus plantarum* CCFM8661 were not thoroughly explored, which may influence their pharmacological effects under different combination conditions. Future research should further investigate these aspects, especially the specific effects of stachyose on the biological characteristics of probiotics. Second, there are certain physiological differences between the intestinal microbiota and immune system of mouse models and humans, which may reduce the general applicability and extrapolation of the experimental results. Studies should validate the practical application of this combination based on the human intestinal microecological environment and immune system.

## 5 Conclusion

The combination of *Lactiplantibacillus plantarum* CCFM8661 and stachyose may enhance immunity through multiple pathways, including mitigating immunosuppressant-induced atrophy of immune organs, intestinal barrier damage, cytokine imbalance, and intestinal microbiota dysbiosis. With the increasing use of probiotics, this study provides valuable insights into the immune-regulating mechanisms of *Lactiplantibacillus plantarum* CCFM8661 combined with stachyose. This research could lead to further optimization and promotion of this therapeutic approach as a novel strategy in the field of immunomodulation. Additionally, this combination may also hold significant potential in anti-inflammatory and intestinal health applications.

## Data Availability

The datasets presented in this study can be found in online repositories. The names of the repository/repositories and accession number(s) can be found below: PRJNA1168472 (SRA).

## References

[1] Liu Y Cao S. The analysis of aerobics intelligent fitness system for neurorobotics based on big data and machine learning. Heliyon. (2024) 10:e33191. doi: 10.1016/j.heliyon.2024.e33191 39022026 PMC11253048

[2] Zhou Y Chu Z Luo Y Yang F Cao F Luo F. Dietary polysaccharides exert anti-fatigue functions via the gut-muscle axis: advances and prospectives. Foods. (2023) 12:3083. doi: 10.3390/foods12163083 37628082 PMC10453516

[3] Rousson V Locatelli I. Years of life lost to COVID-19 and related mortality indicators: an illustration in 30 countries. Biom J. (2024) 66:e202300386. doi: 10.1002/bimj.202300386 39001703 PMC12859533

[4] Francis AI Ghany S Gilkes T Umakanthan S. Review of COVID-19 vaccine subtypes, efficacy and geographical distributions. Postgrad Med J. (2022) 98:389–94. doi: 10.1136/postgradmedj-2021-140654 37066438

[5] Zhang Y Zhou Y Chen J Wu J Wang X Zhang Y. Vaccination shapes within-host SARS-coV-2 diversity of omicron BA. 2.2 Breakthrough Infection. J Infect Dis. (2024) 229:1711–21. doi: 10.1093/infdis/jiad572 38149984

[6] Pan Y Yan J Lu W Shan M. Sub-health status survey and influential factor analysis in chinese during coronavirus disease 2019 pandemic. J Korean Acad Nurs. (2021) 51:5–14. doi: 10.4040/jkan.20241 33706327

[7] Hutchings MI Truman AW Wilkinson B. Antibiotics: past, present and future. Curr Opin Microbiol. (2019) 51:72–80. doi: 10.1016/j.mib.2019.10.008 31733401

[8] Chen X Wei C Zhao J Zhou D Wang Y Zhang S. Carnosic acid: an effective phenolic diterpenoid for prevention and management of cancers via targeting multiple signaling pathways. Pharmacol Res. (2024) 206:107288. doi: 10.1016/j.phrs.2024.107288 38977208

[9] Sun H Feng Y Zhang J Zhang R Ning F She Z. Gastroprotective effects of polysaccharides from purple sweet potato (Ipomoea batatas (L.) Lam) on an ethanol-induced gastric ulcer via regulating immunity and activating the PI3K/Akt/Rheb/mTOR pathway. Food Funct. (2024) 15:6408–23. doi: 10.1039/D4FO01071J 38726829

[10] Li Z Deng Y Sun H Tan C Li H Mo F. Redox modulation with a perfluorocarbon nanoparticle to reverse Treg-mediated immunosuppression and enhance anti-tumor immunity. J Control Release. (2023) 358:579–90. doi: 10.1016/j.jconrel.2023.05.013 37172908

[11] Jeffrey MP Saleem L MacPherson CW Tompkins TA Clarke ST Green-Johnson JM. A Lacticaseibacillus rhamnosus secretome induces immunoregulatory transcriptional, functional and immunometabolic signatures in human THP-1 monocytes. Sci Rep. (2024) 14:8379. doi: 10.1038/s41598-024-56420-8 38600116 PMC11006683

[12] Lee HJ Tran MTH Le MH Justine EE Kim YJ. Paraprobiotic derived from Bacillus velezensis GV1 improves immune response and gut microbiota composition in cyclophosphamide-treated immunosuppressed mice. Front Immunol. (2024) 15:1285063. doi: 10.3389/fimmu.2024.1285063 38455053 PMC10918466

[13] Al-Najjar MAA Abdulrazzaq SB Alzaghari LF Mahmod AI Omar A Hasen E. Evaluation of immunomodulatory potential of probiotic conditioned medium on murine macrophages. Sci Rep. (2024) 14:7126. doi: 10.1038/s41598-024-56622-0 38531887 PMC10965941

[14] Ashique S Mishra N Garg A Kumar N Khan Z Mohanto S. A critical review on the role of probiotics in lung cancer biology and prognosis. Arch Bronconeumol. (2024) 60:S46–58. doi: 10.1016/j.arbres.2024.04.030 38755052

[15] Lao J Yan S Yong Y Li Y Wen Z Zhang X. Lacticaseibacillus casei IB1 alleviates DSS-induced inflammatory bowel disease by regulating the microbiota and restoring the intestinal epithelial barrier. Microorganisms. (2024) 12:1379. doi: 10.3390/microorganisms12071379 39065147 PMC11278699

[16] John D Michael D Dabcheva M Hulme E Illanes J Webberley T. A double-blind, randomized, placebo-controlled study assessing the impact of probiotic supplementation on antibiotic induced changes in the gut microbiome. Front Microbiomes. (2024) 3. doi: 10.3389/frmbi.2024.1359580 PMC12993619 41853507

[17] Medoro A Davinelli S Colletti A Di Micoli V Grandi E Fogacci F. Nutraceuticals as modulators of immune function: A review of potential therapeutic effects. Prev Nutr Food Sci. (2023) 28:89–107. doi: 10.3746/pnf.2023.28.2.89 37416796 PMC10321448

[18] zCristofori F Dargenio VN Dargenio C Miniello VL Barone M Francavilla R. Anti-inflammatory and immunomodulatory effects of probiotics in gut inflammation: A door to the body. Front Immunol. (2021) 12:578386. doi: 10.3389/fimmu.2021.578386 33717063 PMC7953067

[19] Kumar S Ahmad MF Nath P Roy R Bhattacharjee R Shama E. Controlling intestinal infections and digestive disorders using probiotics. J Med Food. (2023) 26:705–20. doi: 10.1089/jmf.2023.0062 37646629

[20] Huaman SOB de Souza FA Bonato MA Dias CP Callegari MA Oba A. Effects of prebiotic and multispecies probiotic supplementation on the gut microbiota, immune function, and growth performance of weaned piglets. PloS One. (2024) 19:e0313475. doi: 10.1371/journal.pone.0313475 39570882 PMC11581253

[21] Jain M Stitt G Son L Enioutina EY. Probiotics and their bioproducts: A promising approach for targeting methicillin-resistant staphylococcus aureus and vancomycin-resistant enterococcus. Microorganisms. (2023) 11:2393. doi: 10.3390/microorganisms11102393 37894051 PMC10608974

[22] Lee NK Park YS Kang DK Paik HD. Paraprobiotics: definition, manufacturing methods, and functionality. Food Sci Biotechnol. (2023) 32:1981–91. doi: 10.1007/s10068-023-01378-y PMC10581967 37860741

[23] Dong J Ping L Zhang K Tang H Liu J Liu D. Immunomodulatory effects of mixed Lactobacillus plantarum on lipopolysaccharide-induced intestinal injury in mice. Food Funct. (2022) 13:4914–29. doi: 10.1039/D1FO04204A 35395665

[24] Chen LH Pan CH Huang SY Chan CH Huang HY. The immunomodulatory effects of long-term supplementation with Lactobacillus casei Shirota depend on ovalbumin presentation in BALB/c mice. Sci Rep. (2021) 11:19478. doi: 10.1038/s41598-021-98791-2 34593870 PMC8484482

[25] Ervina WF Madyawati SP Saputro ID Safari D Putri RE Zulqaida S. A meta-analysis of the effect of probiotic lactobacillus sp. as immunomodulating inflammatory responses. Medeni Med J. (2024) 39:122–31. doi: 10.4274/MMJ.galenos.2024.53822 PMC11572271 38940492

[26] Dong H Wang W Chen Q Chang X Wang L Chen S. Effects of lactoferrin and lactobacillus supplementation on immune function, oxidative stress, and gut microbiota in kittens. Anim (Basel). (2024) 14:1949. doi: 10.3390/ani14131949 PMC11240779 38998061

[27] Goya-Jorge E Gonza I Bondue P Druart G Al-Chihab M Boutaleb S. Unveiling the influence of a probiotic combination of Heyndrickxia coagulans and Lacticaseibacillus casei on healthy human gut microbiota using the TripleSHIME^®^ system. Microbiol Res. (2024) 285:127778. doi: 10.1016/j.micres.2024.127778 38823185

[28] Yu L Zhang L Duan H Zhao R Xiao Y Guo M. The Protection of Lactiplantibacillus plantarum CCFM8661 Against Benzopyrene-Induced Toxicity via Regulation of the Gut Microbiota. Front Immunol. (2021) 12:736129. doi: 10.3389/fimmu.2021.736129 34447391 PMC8383074

[29] Zhai Q Liu Y Wang C Qu D Zhao J Zhang H. Lactobacillus plantarum CCFM8661 modulates bile acid enterohepatic circulation and increases lead excretion in mice. Food Funct. (2019) 10:1455–64. doi: 10.1039/C8FO02554A 30768114

[30] Ma W Lin X Zhao Y Zhang Z Huang L. Protective effect of Lactiplantibacillus plantarum CCFM8661 against heavy metal mixture-induced liver and kidney injury in mice. Food Funct. (2024) 15:6565–77. doi: 10.1039/D4FO01049C 38808610

[31] Li W Zhang S Wang Y Bian H Yu S Huang L. Complex probiotics alleviate ampicillin-induced antibiotic-associated diarrhea in mice. Front Microbiol. (2023) 14:1156058. doi: 10.3389/fmicb.2023.1156058 37125182 PMC10145528

[32] Ta X Wang B Bai J Yu J Chen H Wang C. The source, extraction, purification, physiological function, and application of stachyose in the food industry. Food Chem. (2024) 461:140791. doi: 10.1016/j.foodchem.2024.140791 39163721

[33] Jiang S Li Q Han S Wang H Tang X Wang T. Study on the anti-inflammatory effect of stachyose by inhibiting TLR4/NF-κB signalling pathway *in vitro* and *in vivo* . Clin Exp Pharmacol Physiol. (2023) 50:573–82. doi: 10.1111/1440-1681.13774 36987398

[34] He N Chen K Yu S Cui L Vu SH Jung S. Stachyose exerts anticolitis efficacy by re-balancing treg/th17 and activating the butyrate-derived PPARγ Signaling pathway. J Agric Food Chem. (2024) 72:12171–83. doi: 10.1021/acs.jafc.4c01387 38748640

[35] He L Zhang F Jian Z Sun J Chen J Liapao V. Stachyose modulates gut microbiota and alleviates dextran sulfate sodium-induced acute colitis in mice. Saudi J Gastroenterol. (2020) 26:153–9. doi: 10.4103/sjg.SJG_580_19 PMC7392292 32270772

[36] Liu G Bei J Liang L Yu G Li L Li Q. Stachyose improves inflammation through modulating gut microbiota of high-fat diet/streptozotocin-induced type 2 diabetes in rats. Mol Nutr Food Res. (2018) 62:e1700954. doi: 10.1002/mnfr.201700954 29341443

[37] Wu Y Lu Y Ren D Chen X Yang X. Non-digestive stachyose enhances bioavailability of isoflavones for improving hyperlipidemia and hyperglycemia in mice fed with high fat diet. J Food Drug Anal. (2021) 29:87–97. doi: 10.38212/2224-6614.3078 35696221 PMC9261843

[38] Li W Huang D Gao A Yang X. Stachyose increases absorption and hepatoprotective effect of tea polyphenols in high fructose-fed mice. Mol Nutr Food Res. (2016) 60:502–10. doi: 10.1002/mnfr.201500547 26582073

[39] Wang N Ren D Zhang L Han N Zhao Y Yang X. Effects of sheep whey protein combined with Fu brick tea polysaccharides and stachyose on immune function and intestinal metabolites of cyclophosphamide-treated mice. J Sci Food Agric. (2023) 103:3402–13. doi: 10.1002/jsfa.v103.7 36722467

[40] Park EJ Kim JY Jaiswal V Park HS Ki DB Lee YS. High-molecular-weight Fucoidan exerts an immune-enhancing effect in RAW 264.7 cells and cyclophosphamide-induced immunosuppression rat by altering the gut microbiome. Int Immunopharmacol. (2024) 139:112677. doi: 10.1016/j.intimp.2024.112677 39024753

[41] Ma W Li W Yu S Bian H Wang Y Jin Y. Immunomodulatory effects of complex probiotics on the immuno-suppressed mice induced by cyclophosphamide. Front Microbiol. (2023) 14:1055197. doi: 10.3389/fmicb.2023.1055197 36778877 PMC9911820

[42] Huang H Xie Y Li X Gui F Yang P Li Y. Danggui Buxue decoction regulates the immune function and intestinal microbiota of cyclophosphamide induced immunosuppressed mice. Front Pharmacol. (2024) 15:1420411. doi: 10.3389/fphar.2024.1420411 39224776 PMC11366653

[43] Yan C Qu H Li X Feng B. Holothurian wall hydrolysate ameliorates cyclophosphamide-induced immunocompromised mice via regulating immune response and improving gut microbiota. Int J Mol Sci. (2023) 24:12583. doi: 10.3390/ijms241612583 37628768 PMC10454611

[44] Zheng S Zheng H Zhang R Piao X Hu J Zhu Y. Immunomodulatory effect of ginsenoside rb2 against cyclophosphamide-induced immunosuppression in mice. Front Pharmacol. (2022) 13:927087. doi: 10.3389/fphar.2022.927087 35814238 PMC9263391

[45] Bushmeleva K Vyshtakalyuk A Terenzhev D Belov T Nikitin E Zobov V. Effect of Flavonols of Aronia melanocarpa Fruits on Morphofunctional State of Immunocompetent Organs of Rats under Cyclophosphamide-Induced Immunosuppression. Biomolecules. (2024) 14:578. doi: 10.3390/biom14050578 38785985 PMC11117470

[46] Jung IS Jeon MG Oh DS Jung YJ Kim HS Bae D. Micronized, heat-treated lactobacillus plantarum LM1004 alleviates cyclophosphamide-induced immune suppression. J Med Food. (2019) 22:896–906. doi: 10.1089/jmf.2018.4378 31216204

[47] Kim KJ Paik HD Kim JY. Immune-enhancing effects of lactobacillus plantarum 200655 isolated from korean kimchi in a cyclophosphamide-induced immunocompromised mouse model. J Microbiol Biotechnol. (2021) 31:726–32. doi: 10.4014/jmb.2103.03028 PMC9705930 33820888

[48] Zhang L Liu X Liu Y Cheng X Xu M Qu H. Prebiotics enhance the immunomodulatory effect of Limosilactobacillus fermentum DALI02 by regulating intestinal homeostasis. Food Sci Nutr. (2024) 12:7521–32. doi: 10.1002/fsn3.v12.10 PMC11521649 39479622

[49] You T Zhao Y Liu S Xu H. Lactiplantibacillus plantarum P101 attenuated cyclophosphamide-induced liver injury in mice by regulating the nrf2/ARE signaling pathway. Int J Mol Sci. (2023) 24:13424. doi: 10.3390/ijms241713424 37686229 PMC10488115

[50] Zhou L Yin X Fang B He J Zhan J Zhang X. Effects of Bifidobacterium animalis subsp. lactis IU100 on Immunomodulation and Gut Microbiota in Immunosuppressed Mice. Microorganisms. (2024) 12:493. doi: 10.3390/microorganisms12030493 38543544 PMC10972214

[51] Dong Y-J Lin M-Q Fang X Xie Z-Y Luo R Teng X. Modulating effects of a functional food containing Dendrobium officinale on immune response and gut microbiota in mice treated with cyclophosphamide. J Funct Foods. (2022) 94:105102. doi: 10.1016/j.jff.2022.105102

[52] Zhang N Li C Niu Z Kang H Wang M Zhang B. Colonization and immunoregulation of Lactobacillus plantarum BF_15, a novel probiotic strain from the feces of breast-fed infants. Food Funct. (2020) 11:3156–66. doi: 10.1039/C9FO02745A 32207765

[53] Zeng Y Li T Chen X Fang X Fang C Liang X. Oral administration of Lactobacillus plantarum expressing aCD11c modulates cellular immunity alleviating inflammatory injury due to Klebsiella pneumoniae infection. BMC Vet Res. (2024) 20:399. doi: 10.1186/s12917-024-04248-9 39244529 PMC11380324

[54] Hasan MT Jang WJ Lee BJ Hur SW Lim SG Kim KW. Dietary Supplementation of Bacillus sp. SJ-10 and Lactobacillus plantarum KCCM 11322 Combinations Enhance Growth and Cellular and Humoral Immunity in Olive Flounder (Paralichthys olivaceus). Probiotics Antimicrob Proteins. (2021) 13:1277–91. doi: 10.1007/s12602-021-09749-9 33713023

[55] Nguafack TT Jang WJ Hasan MT Choi YH Bai SC Lee EW. Effects of dietary non-viable Bacillus sp. SJ-10, Lactobacillus plantarum, and their combination on growth, humoral and cellular immunity, and streptococcosis resistance in olive flounder (Paralichthys olivaceus). Res Vet Sci. (2020) 131:177–85. doi: 10.1016/j.rvsc.2020.04.026 32388020

[56] Luo X Zhai Z Lin Z Wu S Xu W Li Y. Cyclophosphamide induced intestinal injury is alleviated by blocking the TLR9/caspase3/GSDME mediated intestinal epithelium pyroptosis. Int Immunopharmacol. (2023) 119:110244. doi: 10.1016/j.intimp.2023.110244 37137263

[57] Yang J Xiao Y Zhao N Pei G Sun Y Sun X. PIM1-HDAC2 axis modulates intestinal homeostasis through epigenetic modification. Acta Pharm Sin B. (2024) 14:3049–67. doi: 10.1016/j.apsb.2024.04.017 PMC11252454 39027246

[58] Oryza SM Wongtangtintharn S Tengjaroenkul B Cherdthong A Tanpong S Pootthachaya P. Investigation of citric acid by-products from rice produced by microbial fermentation on growth performance and villi histology of thai broiler chicken (KKU 1). Vet Sci. (2021) 8:284. doi: 10.3390/vetsci8110284 34822657 PMC8621664

[59] Šefcová MA Santacruz F Larrea-Álvarez CM Vinueza-Burgos C Ortega-Paredes D Molina-Cuasapaz G. Administration of dietary microalgae ameliorates intestinal parameters, improves body weight, and reduces thawing loss of fillets in broiler chickens: A pilot study. Anim (Basel). (2021) 11:3601. doi: 10.3390/ani11123601 PMC8698060 34944376

[60] Rawling M Schiavone M Mugnier A Leclercq E Merrifield D Foey A. Modulation of zebrafish (Danio rerio) intestinal mucosal barrier function fed different postbiotics and a probiotic from lactobacilli. Microorganisms. (2023) 11:2900. doi: 10.3390/microorganisms11122900 38138044 PMC10745996

[61] Ren D Ding M Su J Ye J He X Zhang Y. Stachyose in combination with L. rhamnosus GG ameliorates acute hypobaric hypoxia-induced intestinal barrier dysfunction through alleviating inflammatory response and oxidative stress. Free Radic Biol Med. (2024) 212:505–19. doi: 10.1016/j.freeradbiomed.2024.01.009 38211833

[62] Zeng Z Huang Z Yue W Nawaz S Chen X Liu J. Lactobacillus plantarum modulate gut microbiota and intestinal immunity in cyclophosphamide-treated mice model. BioMed Pharmacother. (2023) 169:115812. doi: 10.1016/j.biopha.2023.115812 37979376

[63] Aljutaily T. Evaluating the nutritional and immune potentiating characteristics of unfermented and fermented turmeric camel milk in cyclophosphamide-induced immunosuppression in rats. Antioxidants (Basel). (2022) 11:792. doi: 10.3390/antiox11040792 35453477 PMC9027126

[64] Akdis M Aab A Altunbulakli C Azkur K Costa RA Crameri R. Interleukins (from IL-1 to IL-38), interferons, transforming growth factor β, and TNF-α: Receptors, functions, and roles in diseases. J Allergy Clin Immunol. (2016) 138:984–1010. doi: 10.1016/j.jaci.2016.06.033 27577879

[65] Delville M Bellier F Leon J Klifa R Lizot S Vinçon H. A combination of cyclophosphamide and interleukin-2 allows CD4+ T cells converted to Tregs to control scurfy syndrome. Blood. (2021) 137:2326–36. doi: 10.1182/blood.2020009187 PMC8163490 33545713

[66] Yoo J Lee J Zhang M Mun D Kang M Yun B. Enhanced γ-aminobutyric acid and sialic acid in fermented deer antler velvet and immune promoting effects. J Anim Sci Technol. (2022) 64:166–82. doi: 10.5187/jast.2021.e132 PMC8819328 35174351

[67] Kang SJ Yang J Lee NY Lee CH Park IB Park SW. Monitoring cellular immune responses after consumption of selected probiotics in immunocompromised mice. Food Sci Anim Resour. (2022) 42:903–14. doi: 10.5851/kosfa.2022.e44 PMC9478974 36133633

[68] Shida K Kiyoshima-Shibata J Nagaoka M Watanabe K Nanno M. Induction of interleukin-12 by Lactobacillus strains having a rigid cell wall resistant to intracellular digestion. J Dairy Sci. (2006) 89:3306–17. doi: 10.3168/jds.S0022-0302(06)72367-0 16899663

[69] Cheng M Shi Y Cheng Y Hu H Liu S Xu Y. Mulberry leaf polysaccharide improves cyclophosphamide-induced growth inhibition and intestinal damage in chicks by modulating intestinal flora, enhancing immune regulation and antioxidant capacity. Front Microbiol. (2024) 15:1382639. doi: 10.3389/fmicb.2024.1382639 38577686 PMC10991686

[70] Xie Z Bai Y Chen G Dong W Peng Y Xu W. Immunomodulatory activity of polysaccharides from the mycelium of Aspergillus cristatus, isolated from Fuzhuan brick tea, associated with the regulation of intestinal barrier function and gut microbiota. Food Res Int. (2022) 152:110901. doi: 10.1016/j.foodres.2021.110901 35181077

[71] Meng Y Wang J Wang Z Zhang G Liu L Huo G. Lactobacillus plantarum KLDS1.0318 ameliorates impaired intestinal immunity and metabolic disorders in cyclophosphamide-treated mice. Front Microbiol. (2019) 10:731. doi: 10.3389/fmicb.2019.00731 31031723 PMC6473033

[72] Zang R Zhou R Li Y Wu H Lu L Xu H. The probiotic Lactobacillus plantarum alleviates colitis by modulating gut microflora to activate PPARγ and inhibit MAPKs/NF-κB. Eur J Nutr. (2024) 64:32. doi: 10.1007/s00394-024-03520-w 39607600

[73] Yang Y Qiao Y Liu G Yi G Liu H Zhang T. Protective effect of a newly probiotic Lactobacillus reuteri LY2-2 on DSS-induced colitis. Eur J Nutr. (2024) 64:5. doi: 10.1007/s00394-024-03535-3 39546032

[74] Alexander M Ang QY Nayak RR Bustion AE Sandy M Zhang B. Human gut bacterial metabolism drives Th17 activation and colitis. Cell Host Microbe. (2022) 30:17–30.e9. doi: 10.1016/j.chom.2021.11.001 34822777 PMC8785648

[75] Marques de Souza PR Keenan CM Wallace LE Habibyan YB Davoli-Ferreira M Ohland C. T cells regulate intestinal motility and shape enteric neuronal responses to intestinal microbiota. Gut Microbes. (2025) 17:2442528. doi: 10.1080/19490976.2024.2442528 39704079 PMC12931703

[76] Xiang XW Zheng HZ Wang R Chen H Xiao JX Zheng B. Ameliorative effects of peptides derived from oyster (Crassostrea gigas) on immunomodulatory function and gut microbiota structure in cyclophosphamide-treated mice. Mar Drugs. (2021) 19:456. doi: 10.3390/md19080456 34436295 PMC8401037

[77] Chen J Zhang C Xia Q Liu D Tan X Li Y. Treatment with subcritical water-hydrolyzed citrus pectin ameliorated cyclophosphamide-induced immunosuppression and modulated gut microbiota composition in ICR mice. Molecules. (2020) 25:1302. doi: 10.3390/molecules25061302 32178470 PMC7144127

[78] Zhang Z Mwizerwa Muhindo E Wang S Yun L Zhang M. Structural characteristics and immunostimulatory activity of sea cucumber tendon polysaccharides in cyclophosphamide-induced Balb/c mice. Food Funct. (2022) 13:8627–42. doi: 10.1039/D2FO00942K 35894650

